# The COVID-19 Sequelae: A Cross-Sectional Evaluation of Post-recovery Symptoms and the Need for Rehabilitation of COVID-19 Survivors

**DOI:** 10.7759/cureus.13080

**Published:** 2021-02-02

**Authors:** Ayman Iqbal, Kinza Iqbal, Shajeea Arshad Ali, Dua Azim, Eisha Farid, Mirza D Baig, Taha Bin Arif, Mohammad Raza

**Affiliations:** 1 Internal Medicine, Dow University of Health Sciences, Karachi, PAK; 2 Medicine, Dow University of Health Sciences, Karachi, PAK; 3 Pediatric Medicine, The Indus Hospital, Karachi, PAK; 4 Pediatric Medicine, Dow University of Health Sciences, Karachi, PAK; 5 Pediatric Medicine, Dr. Ruth K. M. Pfau Civil Hospital, Karachi, PAK

**Keywords:** covid-19, recovery, rehabilitation, quality of life (qol), disease severity, stigma, post-covid-19 symptoms, long-covid, sars-cov-2

## Abstract

Background

As of January 19, 2021, around two million fatalities and 68 million recoveries from coronavirus disease 2019 (COVID-19) have been reported around the globe. The past pandemics of severe acute respiratory syndrome (SARS) and the Middle East respiratory syndrome (MERS) hint toward a risk of occurrence of "Long-COVID" syndrome, i.e., the persistence of post-discharge symptoms among COVID-19 survivors. With the scarcity of literature addressing post-COVID-19 manifestations and little regard for the stigma associated with this disease, survivors' rehabilitation remains widely neglected. The current study aims to assess the prevalence and characteristics of post-COVID-19 manifestations and their effect on the quality of life (QoL) of COVID-19 recovered individuals. We have also analyzed the relationship of time since the recovery of COVID-19 and its severity with the post-discharge symptoms. The stigma affiliated with the infection of SARS coronavirus-2 (SARS-CoV-2) has also been highlighted.

Methodology

A descriptive, cross-sectional, questionnaire-based study was conducted from September 2020 to December 2020 among 158 COVID-19 recovered patients, whose information was obtained from Dow Diagnostic Laboratory, Ojha Campus, Karachi, Pakistan. The questionnaire consisted of four sections: sociodemographic data, post-COVID-19 manifestations, questions relating to the stigma, and the QoL of the recovered COVID-19 patients. We used the EuroQol five-dimension five-level questionnaire to assess the QoL, while the modified BG Prasad Socioeconomic Classification updated for 2019 was employed to determine the socioeconomic status of the participants. Data were analyzed using SPSS version 24.0 (IBM Corp., Armonk, NY, USA). Data were presented in the form of frequencies and percentages.

Results

An overwhelming majority (94.9%) experienced at least one post-COVID-19 symptom, with fatigue (82.9%) being the most prevalent post-discharge manifestation. We observed a significant correlation of post-COVID-19 symptoms with gender, age, and time since recovery. COVID-19 severity was found to be significantly related to the five dimensions of the QoL. A significant difference in EuroQol Visual Analog Scale health score was observed between the participants with mild, moderate, and severe COVID-19 infection (p < 0.001). Besides, the associated stigma with SARS-CoV-2 infection was found to be more prevalent in the participants belonging to the upper class as compared to the other classes (p < 0.05). Nonetheless, we also observed a significant association of disease severity with post-COVID-19 manifestations and pre-existing comorbidities.

Conclusions

The long-COVID syndrome is similar to the post-discharge manifestations of the survivors of prior pandemics of SARS and MERS. Multi-disciplinary rehabilitation teams, healthcare workers, and the general population should recognize the need for systematic assessment of their recovery and further rehabilitation.

## Introduction

Severe acute respiratory syndrome coronavirus-2 (SARS-CoV-2) is the third novel coronavirus identified in the last 18 years to have cross-species transmission [1,2]. With a basic reproduction number (R_o_) of 3.8, the coronavirus disease 2019 (COVID-19) is highly contagious [3]. As of January 19, 2021, almost 95 million global cases with around 68 million recoveries and over two million deaths have been reported. Pakistan alone contributes to nearly 0.5 million of these global cases [4]. COVID-19 symptoms range from fever, cough, and dyspnea in mild cases to severe acute respiratory syndrome (SARS) and respiratory failure in critically ill patients requiring hospitalization [5]. Respiratory symptoms may also be accompanied by cardiac, gastrointestinal, renal, hepatic, neurological, cutaneous, hematological, olfactory, and gustatory manifestations [6].

The number of COVID-19 survivors being discharged from the hospital is increasing every day; however, to date, very little evidence is available on the long-term effects of COVID-19 faced by survivors following hospital discharge. An Italian study revealed that 32% of the previously hospitalized patients complained of the persistence of one or two COVID-19-associated symptoms, while 55% reported three or more symptoms even two months after being discharged [7]. Of these post-COVID-19 symptoms, fatigue (53.1%) and dyspnea (43.4%) were the most widely reported [7]. The prevalence of these symptoms, also known as "Long-COVID," is in alignment with the post-discharge manifestations experienced by the survivors of the SARS outbreak in 2002 and the Middle East respiratory syndrome (MERS) outbreak in 2012 [8,9]. SARS and MERS survivors suffered not only from physical ramifications (reduced exercise capacity and lung function) but also from mental health impairment (anxiety and depression) [9]. Similarly, owing to the stigma associated with COVID-19, the survivors face constant shaming and discrimination, adding a psychological component to their physical suffering [10]. However, with most current literature focused on the acute management of COVID-19, data regarding post-COVID-19 manifestations are sparse.

The current study aims to assess the prevalence and characteristics of post-COVID-19 manifestations and determine the correlations, if any, with factors like gender, age, and disease severity. We also aim to investigate the stigma associated with being a COVID-19 survivor and explore probable post-COVID-19 rehabilitation strategies for the hospital- and home-treated COVID-19 patients.

## Materials and methods

Study design and participants

A cross-sectional, questionnaire-based study was conducted in Karachi, Pakistan, among 158 recovered patients of COVID-19. The patients’ data were obtained from the Dow Diagnostic Laboratory, Ojha Campus, between September 2020 and December 2020. Our study included COVID-19 survivors with a negative-reverse transcription-polymerase chain reaction (RT-PCR) for the last 20-90 days. Participants >18 years of age from either gender were selected by convenience sampling technique. We excluded any COVID-19 patient who was not tested positive and/or negative by an RT-PCR. Moreover, any COVID-19 survivors with a history of psychiatric illness were also excluded from the study.

We calculated the sample size through the OpenEpi.com sample size calculator version 3.01 [11]. Using an anticipated frequency (p) of 89.2%, a confidence interval (CI) of 95%, and a 5% degree of precision, the estimated sample size was 149 [12]. In order to obtain the maximum responses, a total of 200 participants were approached. After excluding the incomplete interviews, 158 responses were included in the study. The response rate was, therefore, 79.0%.

Study tool

The questionnaire was designed after a comprehensive review of literature from the past studies based on similar objectives [10,12]. A pilot study was carried out before the survey, recruiting 20 COVID-19 recovered patients >18 years of age for the questionnaire-based interviews. We used the feedback of those patients to modify and refine the questionnaire. The questionnaire was finalized with the help of the expert opinion of a senior consultant. 

The final questionnaire consisted of 24 questions; the questionnaire was subdivided into four sections. There were three open-ended and 21 close-ended questions. The first section covered the sociodemographic data of the participants, such as age, gender, educational level, employment status, place of residence, family income per month, and comorbidities. The second section inquired about the post-COVID-19 manifestations including, fatigue, depression, dyspnea, persistent cough, joint pain, persistent headache, poor sleep, and others. The third section assessed the stigma associated with being a COVID-19 recovered individual. For this purpose, we used three questions from the stigma questionnaire by Dar et al., consisting of a total of 15 items [10]. A 4-point Likert scale (1: strongly disagree, 2: disagree, 3: agree, 4: strongly agree) was used to score each of these questions [13]. The final section evaluated the quality of life (QoL) after recovery from COVID-19 by using the EuroQol five-dimension five-level questionnaire (EQ-5D-5L) telephone interview version. The EQ-5D-5L measures the QoL based on a five-component scale (mobility, self-care, usual activities, pain/discomfort, and anxiety/depression) [14]. Each component has five response levels: no problems (Level 1), slight problems (Level 2), moderate problems (Level 3), severe problems (Level 4), and extreme problems (Level 5). 

The modified BG Prasad Socioeconomic Classification updated for 2019 was deployed to assess the socioeconomic status of the subjects [15]. Using relevant data, we classified the participants according to this scale as upper, upper-middle, middle, lower-middle, and lower classes.

Data collection

The recovered COVID-19 patients were contacted with the help of the information obtained from the Dow Diagnostic Laboratory. After explaining the purpose of the study, informed verbal consent was taken from each participant. Questionnaire-based interviews were conducted via telephone- and video-calls. The questionnaire was translated into the lingua franca, Urdu, and investigators conducted one-on-one interviews to assist and facilitate the participants. We made multiple calls at different times of the day to ensure maximum responses. The respondents were free to withdraw from the study at any point. Confidentiality and anonymity were maintained throughout the data collection and investigation. 

Statistical analysis

Data were analyzed using Statistical Package for Social Sciences (SPSS) version 24.0 (IBM Corp., Armonk, NY, USA). Descriptive statistics, such as frequencies, percentage, and mean and standard deviation (SD), were employed for the presentation of categorical and continuous variables, respectively. The independent-samples t-test and one-way analysis of variance (ANOVA) were applied to analyze continuous variables, while the chi-square test was used for categorical variables. A p-value of <0.05 was considered statistically significant.

## Results

The baseline characteristics such as the sociodemographic status, pre-COVID-19 comorbidities, and COVID-19 course of the 158 respondents are listed in Table 1. In brief, the mean age of the study sample was 32.10 ± 12.42 years (age range: 19-80 years). At the time of the study, the participants’ mean time since recovery was 38.10 ± 20.00 days. Most of the respondents in our study were females (55.1%). Approximately 40% of the study population reported pre-existing comorbidities; hypertension (13.3%) was the leading comorbidity, followed by asthma (10.1%), diabetes mellitus (9.5%), and cardiovascular diseases (7%). The clinical course of COVID-19 was classified based on the severity of the symptoms into three groups of mild, moderate, and severe; respondents who managed with home remedies (70.9%) were regarded as having mild symptoms, those who received oxygen therapy (20.9%) had moderate clinical course, while those admitted in an intensive care unit (ICU) had severe COVID-19 infection.

**Table 1:** Sociodemographics and clinical characteristics of the study sample (N = 158) SD: standard deviation; ICU: intensive care unit

Characteristics	Value, n (%)
Age (years), mean ± SD	40.10 ± 12.42
Gender	
Female	87 (55.1)
Male	71 (44.9)
Education	
Primary	10 (6.3)
Secondary	9 (5.7)
Intermediate	32 (20.3)
Graduate	58 (36.7)
Post-graduate	49 (31.0)
Occupation	
Student	38 (24.1)
Housewife	29 (18.4)
Job	65 (41.1)
Business	16 (10.1)
Unemployed/retired	10 (6.3)
Residence	
Urban	132 (83.5)
Rural	26 (16.5)
Socioeconomic classes	
Upper	129 (81.6)
Upper-middle	2 (1.3)
Middle	10 (6.3)
Middle-lower	10 (6.3)
Lower	7 (4.4)
Do you smoke?	
Yes	19 (12.0)
No	139 (88.0)
Comorbidities	
Diabetes mellitus	15 (9.5)
Hypertension	21 (13.3)
Asthma	16 (10.1)
Hypothyroidism	5 (3.2)
Chronic kidney disease	2 (1.3)
Cardiovascular disease	11 (7.0)
Other conditions	27 (17.1)
Time since recovery to study entry (days), mean ± SD	38.10 ± 20.00
Severity of disease	
Mild (manage at home by remedies)	112 (70.9)
Moderate (oxygen therapy used only)	33 (20.9)
Severe (ICU)	13 (8.2)

Upon analyzing long-term COVID-19 manifestations, we found that an overwhelming majority (94.9%) experienced at least one post-COVID-19 symptom. The vast majority of the population complained of persistent fatigue (82.9%), poor sleep quality (56.3%), anxiety (53.2%), and dyspnea (50%). COVID-19-related symptoms post-recovery are listed in Table 2. 

**Table 2:** Characteristics of post-COVID-19 manifestations and quality of life of participants (N = 158) SD: standard deviation; COVID-19: coronavirus disease 2019

Characteristics	Value, n (%)
Post-COVID-19 manifestations	
Fatigue	131 (82.9)
Poor sleep quality	89 (56.3)
Anxiety	84 (53.2)
Dyspnea	79 (50.0)
Joint pain	75 (47.5)
Loss of smell and taste	75 (47.5)
Persistent cough	70 (44.3)
Depression	67 (42.4)
Hair fall	63 (39.9)
Continuous headache	57 (36.1)
Chest pain	56 (35.4)
Intermittent fever	54 (34.2)
Brain fog	30 (19.0)
Blurred vision	30 (19.0)
Tinnitus	30 (19.0)
Pulmonary fibrosis	6 (3.8)
Renal failure	2 (1.3)
Stroke	1 (6.0)
Diabetes	1 (6.0)
Did you go for any extra investigations because of the post-COVID-19 manifestations?	
Yes	58 (36.7)
No	100 (63.3)
Did you receive medications for post-COVID-19 manifestations?	
Yes	83 (52.5)
No	75 (47.5)
Did your condition improve on treatment?	
Yes	70 (84.3)
No	13 (15.7)
Quality of life	
After recovery, were you able to walk about?	
Yes, without any problems	79 (50)
Yes, with slight problems	52 (32.9)
Yes, with moderate problems	22 (13.9)
Yes, with severe problems	4 (2.5)
No, I was unable to walk	1 (0.6)
After recovery, were you able to wash or dress yourself?	
Yes, without any problems	110 (69.6)
Yes, with slight problems	30 (19.0)
Yes, with moderate problems	13 (8.2)
Yes, with severe problems	3 (1.9)
No, I was unable to wash or dress myself	2 (1.3)
After recovery, were you able to carry out your usual activities? (business, office work, study, housework or leisure activities)	
Yes, without any problems	43 (27.2)
Yes, with slight problems	45 (28.5)
Yes, with moderate problems	42 (26.6)
Yes, with severe problems	18 (11.4)
No, I was unable to carry out my usual activities	10 (6.3)
After recovery, did you feel pain or discomfort?	
No pain or discomfort	41 (25.9)
Slight pain or discomfort	73 (46.2)
Moderate pain or discomfort	30 (19.0)
Severe pain or discomfort	12 (7.6)
Extreme pain or discomfort	2 (1.3)
After recovery, were you anxious or depressed?	
Not anxious or depressed	63 (39.9)
Slightly anxious or depressed	54 (34.2)
Moderately anxious or depressed	18 (11.4)
Severely anxious or depressed	16 (10.1)
Extremely anxious or depressed	7 (4.4)
On a scale of 0-100 how good or bad your health is today? (100 being the best possible health you can imagine), mean ± SD	70.76 ± 22.42

Moreover, we found a statistically significant relation of age with the presence of post-COVID-19 manifestations such as dyspnea (p = 0.007), a persistent cough (p < 0.001), joint pain (p < 0.001), and chest pain (p < 0.001). Interestingly, a significantly higher number of females suffered from persistent COVID-19-associated symptoms, such as fatigue (58.8%), anxiety (66.7%), joint pain (64%), continuous headache (73.7%), depression (67.2%), and hair fall (74.6%) as compared to their counterparts (p < 0.05).

We observed that 52.5% of our study participants received medications for the post-recovery symptoms, and out of these respondents, around 84.3% reported an improvement in their condition. Notably, we found a statistically significant association between COVID-19 severity and the extra investigations sought post-recovery (p < 0.001).

As seen in Table 2, we asked a series of questions to predict the effect of COVID-19 on the QoL of its survivors. The severity of COVID-19 was found to be significantly (p < 0.05) related to five dimensions of EQ-5D-5L, i.e., problems in carrying out usual activities, difficulty in washing or dressing oneself, difficulty in walking, pain, and discomfort, and anxiety and depression. Of severely affected subjects, 77% experienced either severe problems or were unable to carry out their usual activities. On the other hand, 75% of mildly affected patients reported either no or slight problems in their day-to-day activities.

According to the EuroQol Visual Analog Scale (EQ-VAS) health score, the mean health score of the respondents was 70.76 ± 22.42. There was a statistically significant association between the infection severity and the EQ-VAS health score (p < 0.001). The mean difference of score between mild and moderately infected patients was 29.93 ± 3.20, while that between mild and severely infected patients was 42.84 ± 4.74 (one-way analysis of variance).

**Figure 1:**
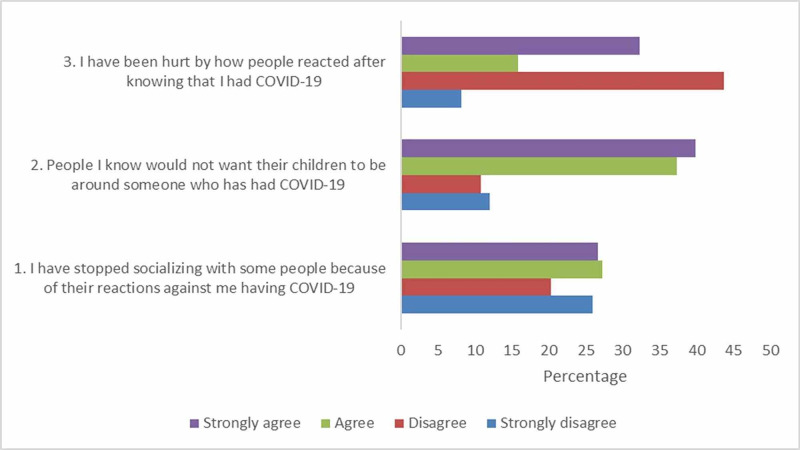
Stigma associated with COVID-19 COVID-19: coronavirus disease 2019

We observed a significant relation (p < 0.05) between the socioeconomic classes and the stigma associated with recovery from COVID-19. Interestingly, the stigma associated with COVID-19 survivors was more prevalent in the upper class than the lower- and lower-middle class. When asked about reducing contact with people who reacted inconsiderately to their diagnosis, all participants from the lower and lower-middle class opted either to disagree or strongly disagree. The majority of the participants (80%) from the middle and lower-middle class did not believe that others would want to keep their children away from COVID-19 survivors. Figure 1 represents the stigma associated with being a COVID-19 survivor.

As shown in Table 3, a statistically significant (p < 0.05) relation was observed between time since recovery from COVID-19 and post-recovery symptoms (fatigue, dyspnea, persistent cough, joint pain, continuous headache, poor sleep quality, chest pain, and intermittent fever). Moreover, in our study, no significant relation (p > 0.05) was found between time since recovery and other post-COVID-19 manifestations including anxiety, depression, hair fall, brain fog, tinnitus, pulmonary fibrosis, and loss of taste and smell. More than half (63%) of moderate-to-severely affected COVID-19 patients reported at least one comorbidity. A significant relation (p < 0.05) of COVID-19 severity was observed with post-COVID-19 symptoms (fatigue, anxiety, dyspnea, persistent cough, joint pain, poor sleep quality, depression, chest pain, hair fall, brain fog, tinnitus, stroke, and renal failure) and comorbidities (diabetes mellitus, hypertension, cardiovascular disease, and chronic kidney disease), as demonstrated in Table 4.

**Table 3:** Association of post-COVID-19 manifestations with time since recovery from COVID-19 (N = 158) Calculated using independent-samples t-test; *p-value of <0.05 considered statistically significant SD: standard deviation; COVID-19: coronavirus disease 2019

Post-COVID-19 manifestations	Time since recovery (days) mean ± SD	p-Value
Fatigue		<0.001*
Yes	33.98 ± 15.62	
No	58.07 ± 26.37	
Anxiety		0.241
Yes	36.34 ± 19.31	
No	40.09 ± 20.70	
Dyspnea		<0.001*
Yes	30.92 ± 11.92	
No	45.28 ± 23.64	
Persistent cough		<0.001*
Yes	30.59 ± 11.45	
No	44.08 ± 23.16	
Joint pain		0.001*
Yes	32.59 ± 15.28	
No	43.08 ± 22.41	
Continuous headache		0.028*
Yes	33.47 ± 14.70	
No	40.71 ± 22.09	
Poor sleep quality		<0.001*
Yes	32.81 ± 13.28	
No	44.93 ± 24.73	
Chest pain		<0.001*
Yes	30.37 ± 10.51	
No	42.34 ± 22.59	
Depression		0.664
Yes	38.91 ± 21.04	
No	37.51 ± 19.29	
Hair fall		0.946
Yes	37.97 ± 20.32	
No	38.19 ± 19.90	
Loss of smell and taste		0.371
Yes	36.60 ± 18.82	
No	39.46 ± 21.03	
Brain fog		0.731
Yes	36.97 ± 19.20	
No	38.37 ± 20.25	
Intermittent fever		0.001*
Yes	31.15 ± 13.89	
No	41.71 ± 21.72	
Blurred vision		0.864
Yes	38.67 ± 22.27	
No	37.97 ± 19.52	
Tinnitus		0.195
Yes	33.83 ± 17.25	
No	39.10 ± 20.53	
Pulmonary fibrosis		0.813
Yes	40.00 ± 22.36	
No	38.03 ± 19.98	
Diabetes		0.956
Yes	37.00 ± 0.00	
No	38.12 ± 20.06	
Stroke		0.481
Yes	24.00 ± 0.00	
No	38.19 ± 20.03	
Renal failure		0.799
Yes	34.50 ± 0.71	
No	38.15 ± 20.12	

**Table 4:** Correlation of severity of disease with comorbidities and post-COVID-19 manifestations (N = 158) Calculated using chi-square test; *p-value of <0.05 considered statistically significant COVID-19: coronavirus disease 2019

	Mild, n (%)	Moderate, n (%)	Severe, n (%)	p-Value
Comorbidities				
Diabetes mellitus	4 (26.7)	5 (33.3)	6 (40.0)	<0.001*
Hypertension	6 (28.6)	7 (33.3)	8 (38.1)	<0.001*
Asthma	10 (62.5)	4 (25.0)	2 (12.5)	0.699
Hypothyroidism	3 (60.0)	2 (40.0)	0 (0.0)	0.493
Chronic kidney disease	0 (0.0)	1 (50.0)	1 (50.0)	0.038*
Cardiovascular disease	4 (36.4)	3 (27.3)	4 (36.4)	0.001*
Other conditions	15 (55.6)	9 (33.3)	3 (11.1)	0.148
Post-COVID-19 manifestations				
Fatigue	86 (65.6)	33 (25.2)	12 (9.2)	0.005*
Anxiety	49 (58.3)	26 (31.0)	9 (10.7)	0.001*
Dyspnea	39 (49.4)	28 (35.4)	12 (15.2)	<0.001*
Persistent cough	35 (50.0)	26 (37.1)	9 (12.9)	<0.001*
Joint pain	42 (56.0)	21 (28.0)	12 (16.0)	<0.001*
Continuous headache	36 (63.2)	16 (28.0)	5 (8.8)	0.225
Poor sleep quality	55 (61.8)	25 (28.1)	9 (10.1)	0.016*
Chest pain	19 (33.9)	25 (44.6)	12 (21.4)	<0.001*
Depression	38 (56.7)	22 (32.8)	7 (10.4)	0.003*
Hair fall	42 (66.7)	19 (30.1)	2 (3.2)	0.020*
Loss of smell and taste	54 (72.0)	16 (21.3)	5 (6.7)	0.794
Brain fog	15 (50.0)	11 (36.7)	4 (13.3)	0.020*
Intermittent fever	33 (61.1)	15 (27.8)	6 (11.1)	0.150
Blurred vision	18 (60.0)	8 (26.7)	4 (13.3)	0.304
Tinnitus	16 (53.3)	11 (36.7)	3 (10.0)	0.046*
Pulmonary fibrosis	3 (50.0)	1 (16.7)	2 (33.3)	0.074
Diabetes	0 (0.0)	1 (100.0)	0 (0.0)	0.149
Stroke	0 (0.0)	0 (0.0)	1 (100.0)	0.004*
Renal failure	0 (0.0)	1 (50.0)	1 (50.0)	0.038*

## Discussion

With the sudden rise in the number of active cases, Pakistan has been hit by the second wave of COVID-19 [16]. Although the number of patients recovering from COVID-19 is rising every day, the post-recovery symptoms are poorly documented in the literature. The recent literature primarily focuses on the prognosis of COVID-19, the susceptibility to infection, and the impact of the current pandemic on the healthcare system. The rehabilitation of recovered patients, therefore, remains largely unaddressed. The present study addresses the paucity of literature regarding the post-COVID-19 symptoms and their impact on the QoL of recovered individuals. Moreover, it sheds light on the importance of establishing a multi-disciplinary rehabilitation team of physicians, nurses, psychiatrists, psychologists, mental health counselors, and occupational therapists to deliver personalized care to the survivors.

In our study, we observed a statistically significant relationship between the severity of COVID-19 and post-COVID-19 symptoms. Our results are consistent with the finding by Kamal et al., which revealed that Egyptian subjects with severe COVID-19 had more severe post-recovery manifestations than those with milder disease [12]. In alignment with the follow-up studies conducted in Italy, the United Kingdom (UK), and Egypt, fatigue was found to be the most prevalent post-COVID-19 manifestation in our study [7,9,12]. In addition, post-recovery fatigue is also common following infection with Coxsackie B viruses, arboviruses, human herpes virus-6, Ebstein Barr virus, and cytomegalovirus [17]. Likewise, previous outbreaks of SARS and MERS also showed a similar trend [18]. Evidence suggests that some patients developed chronic fatigue syndrome/myalgic encephalomyelitis (CFS/ME) after the acute SARS infection. CFS/ME encompasses persistent fatigue, diffuse muscle pain, depression, and sleep disturbances [18]. Ahmed et al. conducted a meta-analysis of follow-up studies of recovered SARS and MERS patients; fatigue was reported in at least one-third of the patients in two studies with a follow-up period of 18 and 40 months, respectively [19]. Furthermore, dyspnea was another common post-COVID-19 manifestation reported in the present study. The presence of dyspnea post-recovery could be explained by the fact that SARS survivors experience respiratory compromise owing to abnormalities in diffusion lung capacity of carbon monoxide, forced vital capacity, and total lung capacity even at six months post-discharge [19].

We observed a statistically significant (p < 0.05) relation between time since recovery from COVID-19 and post-recovery symptoms (fatigue, dyspnea, etc.). In line with our results, a follow-up study conducted in London revealed higher breathlessness scores in discharged patients who got assessed earlier, indicating that dyspnea may subside gradually [8]. Interestingly, our study showed no significant relationship between the time since recovery and other post-COVID-19 manifestations, including anxiety, depression, hair fall, brain fog, tinnitus, pulmonary fibrosis, and loss of taste and smell (p > 0.05). This suggests that these manifestations might persist long after recovery, possibly requiring rehabilitation.

Our participants also reported neurological manifestations such as brain fog, tinnitus, poor sleep quality, depression, and anxiety. Around half (56.3%) of our study subjects complained of poor sleep quality. Similarly, post-SARS patients also complained of altered sleep habits. It is suspected that the pathophysiology of disturbed post-infection sleep quality in SARS-CoV-2 may be similar to SARS-CoV-1, owing to the structural and clinical similarities [20]. Around 19% of our study participants reported brain fog. An observational cross-sectional study also revealed that the recently recovered patients from COVID-19 had substantial cognitive impairment compared to healthy control subjects, which could be due to the underlying inflammation. COVID-19 subjects had a slower reaction time and a lower score on online neuropsychological tests gauging continuous and selective attention [21]. Surprisingly, 19% of our study population complained of tinnitus. In Qatar, a patient with mild COVID-19 experienced tinnitus during the infection and even after recovery. Hence, this case report highlighted the importance of detailed audiological evaluation in patients who experience isolated tinnitus and hearing loss during the COVID-19 infection and after recovery [22].

Our results revealed that a significantly higher number of females developed post-COVID-19 hair fall, anxiety, depression, and fatigue when compared to males (p < 0.05). According to dermatologists, telogen effluvium is a temporary form of hair loss induced by stress, high fever, or infection. As there is a two- to three-month lag between the stressful event and onset of telogen effluvium, most patients report this condition post-recovery. Reassurance and counseling regarding the self-limitation of telogen effluvium can preclude the incidence of anxiety and depression in women suffering from this condition. If the hair loss persists beyond six to nine months, biotin supplementation and 5% Minoxidil is recommended [23]. Almost half (47.5%) of our study participants complained of loss of taste and smell post-recovery. Over 90% of COVID-19 patients regain their gustatory and olfactory sensation within the first month. However, if the loss of taste and smell persists beyond a month, it can be alarming since the most common cause of permanent olfactory loss is post-viral anosmia. Although the exact mechanism of post-infectious anosmia is unknown, it may be due to infection of the olfactory bulb by SARS-CoV-2 through the pathway of olfactory neurons [24]. There is no definite pharmacological treatment for post-viral loss of smell. Depending upon the severity and the duration of loss of smell, olfactory training is a viable option to regain the sense of smell [25].

After recovery, the COVID-19 survivors often experience discrimination and prejudice because of the community’s irrational fear that they are still contagious. Minimizing social interaction with the COVID-19 survivors to the point that they feel hurt is an expression of enacted stigma. Our results demonstrated a significant relationship between socioeconomic classes and the stigma associated with being a COVID-19 survivor (p < 0.05). We noticed that the upper-class subjects were comparatively more stigmatized than the lower- and lower-middle-class participants. A cross-sectional exploratory study by Dar et al. also reported similar findings. As individuals from upper socioeconomic classes are more likely to be employed and engage in public interaction, there is a higher probability of facing stigma. Survivors from prior infectious epidemics had to encounter similar experiences immediately after being reintegrated into society [10].

There was a statistically significant relationship between disease severity and the EQ-VAS health score (p < 0.001). The mean difference of score between mild and severely infected patients was 42.84 ± 4.74. Moreover, the severity of COVID-19 was significantly related to the five dimensions of EQ-5D-5L (p < 0.05). Approximately three-quarters (77%) of severely ill patients experienced either severe problems or were unable to carry out their usual activities. In concordance with this, a follow-up study conducted in post-discharge patients in the UK showed a clinically significant drop in EQ-5D-5L in 68.8% of ICU patients and 45.6% of subjects in the ward group [9]. Survivors of previous outbreaks such as SARS and MERS had considerably reduced health-related QoL as measured by SF-36, even six months post-recovery. Tansey et al. reported that 17% of SARS survivors were not able to return to their prior working-level even one year after infection [26].

The association between the severity of COVID-19 and the extra investigations carried out post-recovery was also found to be statistically significant (p < 0.001). A follow-up computed tomography (CT) scan can identify residual pulmonary fibrosis. Different stages of gradual recovery from ground-glass opacities to consolidation and to complete resolution can be monitored via CT scans [27]. In the previous epidemics, CT scan from recovered patients showed that pulmonary fibrosis can persist for up to seven years, thus highlighting the importance of follow-up imaging to identify residual lung fibrosis at an early stage and offering rehabilitation to improve long-term outcomes [19]. Only 52.5% of our study participants received medications for post-COVID-19 symptoms. Out of these patients, 84.3% reported an improvement in their condition. This highlights how proper consultation can help in early recuperation.

Although the negative PCR for COVID-19 is celebratory, the survivors' functional return is multi-dimensional and time-taking. The recovery needs of home-managed, mild COVID-19 patients remain considerably sidelined and unaddressed, in contrast to hospitalized patients. In a developing country like Pakistan, the first step is to recognize the heterogeneity of post-COVID-19 manifestations and create awareness among the general population. Healthcare workers and rehabilitation teams should teach the general public to empathize with their fellow recovered COVID-19 survivors and help them gradually return to normalcy [28]. The potential role of media in addressing the stigma surrounding the SARS-CoV-2 infection cannot be undermined [29]. For mild post-COVID-19 manifestations such as fatigue, supportive treatment includes vitamins, adequate hydration, and home-based monitoring of the patients [28]. Counseling, yoga, and breathing exercises can help to counter anxiety and depression. It is important to dispel any myths reinforcing the idea that a COVID-19 survivor is still contagious after recovery to help reduce the stigma and allow rapid re-integration of the individual into society. Factual information regarding COVID-19 should be made available via media and healthcare workers, which can cease the emergence of any upcoming fledgling myths in the society. Moreover, extra consideration should be given to improve the mental well-being and raising the morale of frontline workers since the ratio of infectivity is generally higher among that population [29]. Finally, the recovered subjects should be highly vigilant in maintaining and monitoring their health status as there is a risk of future complications after recovery.

Limitations

Our study has certain limitations. First, it is a single-center study with limited coverage of socioeconomic classes; the majority of the participants belonged to the upper class. More versatile studies should be carried out in the future involving a bigger sample size and a more diverse population to assess the health of COVID-19 survivors. Secondly, the sample population was not randomized, and there may have been a recall bias in answering some questions in the survey. Thirdly, patients requiring prolonged ICU and inpatient stay might be under-represented in this study. Fourth, out of the 15 questions of the stigma questionnaire, we included only three questions in our questionnaire. Adding all 15 questions would have prolonged our questionnaire, which could have been cumbersome for our study participants. Finally, since this is a questionnaire-based survey, social desirability bias could not be excluded. 

## Conclusions

Most of the COVID-19 survivors experienced mild post-recovery symptoms such as fatigue, poor sleep quality, and anxiety, and a few reported more severe manifestations such as pulmonary fibrosis, renal failure, diabetes mellitus, and stroke. The presence of post-COVID-19 manifestations is related to comorbidities and disease severity. The long-COVID syndrome is similar to post-discharge symptoms experienced by survivors of prior pandemics of SARS and MERS. Multi-disciplinary rehabilitation teams, healthcare workers, and the general population should recognize the need for systematic assessment of their recovery and further rehabilitation. Similar follow-up studies should be conducted in the future to investigate the lingering effects of COVID-19 and devise strategies to deal with the aftermath.
